# A commentary on “Development and validation of a machine learning-based risk model for metastasis in patients with nmCRPC”

**DOI:** 10.1097/JS9.0000000000002966

**Published:** 2025-07-07

**Authors:** Han Li, Ming Liu, Miao Wang

**Affiliations:** Department of Urology, Beijing Hospital, National Center of Gerontology, Institute of Geriatric Medicine, Chinese Academy of Medical Sciences & Peking Union Medical College, Beijing, China

*Dear Editor,*

We read with great interest the article by Ni *et al*^[1]^, titled “Development and Validation of a Machine Learning-Based Risk Model for Metastasis in Patients with nmCRPC,” published in the *International Journal of Surgery*. The authors have developed a robust machine learning-driven prognostic model utilizing data from SPARTAN and ARAMIS phase III clinical trials. By integrating key clinical variables such as the use of novel hormone therapies, PSA doubling time, and hemoglobin levels, the model significantly improves its relevance and applicability in clinical practice. The model demonstrated strong discriminatory performance, offering a valuable tool for predicting metastasis-free survival in nonmetastatic castration-resistant prostate cancer (nmCRPC) patients. No artificial intelligence tools were used in the writing or analysis of this manuscript, in accordance with the TITAN Guidelines 2025^[2]^.

Patients with nmCRPC are at high risk of progression, but traditional PSA-based markers offer limited predictive value. Accurate prognostic models are essential for guiding personalized treatment and improving survival. While this study offers valuable insights, further methodological and clinical refinement may improve its applicability.

First, the model was trained solely on the SPARTAN cohort and validated on the ARAMIS cohort. However, SPARTAN included only high-risk nmCRPC patients with PSADT ≤10 months, whereas ARAMIS enrolled a broader population, including those with PSADT >10 months^[3,4]^. Notably, nodal status differed markedly between the cohorts: 83.5% of patients in SPARTAN were N0 and 16.5% were N1, while in ARAMIS, only 49.5% were N0, with 16.5% N1 and 34.0% NX (unknown). The high proportion of NX cases in the validation cohort introduces uncertainty regarding disease burden and may compromise the reliability of external validation. The authors did not perform propensity score matching or stratified analyses to adjust for these baseline imbalances. Thus, the model’s predictive accuracy requires further validation in subgroups such as patients with PSADT >10 months, unknown nodal status, or distinct treatment backgrounds.

Second, the study’s approach to variable classification may have limited its analytical depth. Race was simplified into a binary variable (White vs. non-White), which fails to capture the distinct clinical and socioeconomic characteristics of subgroups such as Black or Asian patients. Similarly, details regarding prior treatment strategies were not clearly delineated, making it difficult to assess their potential influence on outcomes. Furthermore, although lactate dehydrogenase (LDH) is a well-recognized prognostic indicator in nmCRPC, it was omitted from the model due to missing data, and no imputation methods were employed to address this limitation.

Third, although the authors explored various machine learning approaches, the final model ultimately relied on a linear combination of LASSO and Cox regression. Notably, the more sophisticated Random Survival Forest (RSF) algorithm demonstrated excellent discrimination in the training cohort (C-index = 0.94), but its performance declined substantially upon external validation. This underscores a key point: more complex models are prone to overfitting and may not generalize well across different patient populations.

Additionally, the study did not evaluate whether the multivariable model offered incremental value over PSADT stratification alone. No direct comparison was made between a PSADT-only model and the final model. The lack of exploration into interaction effects between PSADT and other variables further limits clinical utility assessment.

Finally, external validation was limited to clinical trial cohorts, without support from real-world populations. Moreover, the omission of PSMA-PET, limits the model’s clinical relevance^[5]^.

In conclusion, this study represents one of the first externally validated machine learning-based prognostic models for predicting metastasis-free survival in patients with nmCRPC. Unlike previous approaches that largely relied on single indicators such as PSADT, this model integrates multiple clinical variables into a comprehensive risk score, enhancing predictive accuracy. The findings underscore the importance of external validation across different trial populations and highlight the potential value of refined staging definitions and incorporation of emerging markers like PSMA-PET imaging. These insights provide practical guidance for individualized patient management and future clinical trial designs.

## Data Availability

Due to the nature of this submission as a correspondence, no new data have been collected in its creation or writing.
